# Sensitivity and Specificity of Ultrawide-Field Fundus Photography for the Staging of Sickle Cell Retinopathy in Real-Life Practice at Varying Expertise Level

**DOI:** 10.3390/jcm8101660

**Published:** 2019-10-11

**Authors:** Roxane Bunod, Alexandra Mouallem-Beziere, Francesca Amoroso, Vittorio Capuano, Karen Bitton, Cynthia Kamami-Levy, Camille Jung, Eric H. Souied, Alexandra Miere

**Affiliations:** 1 Department of Ophthalmology, Centre Hospitalier Intercommunal de Creteil University Paris Est Creteil, 40 Avenue de Verdun, 94000 Creteil, France; 2 Clinical Research Center, Centre Hospitalier Intercommunal de Creteil University Paris Est Creteil, 40 Avenue de Verdun, 94000 Creteil, France

**Keywords:** sickle cell retinopathy, ultrawide-field imaging, ultrawide-field fundus photography, ultrawide-field angiography

## Abstract

Purpose: To evaluate the sensitivity and specificity of ultrawide-field fundus photography (UWF-FP) for the detection and classification of sickle cell retinopathy (SCR) by ophthalmologists with varying degrees of expertise in retinal disease. Methods: Patients presenting with sickle cell disease (SCD) in the Créteil University Eye Clinic, having undergone UWF-FP and ultrawide-field fluorescein angiography (UWF-FA) on the same day, were retrospectively included. Eyes with previous retinal photocoagulation were excluded. SCR was graded independently by UWF-FP and UWF-FA using Goldberg classification by two ophthalmologists with varying expertise levels. Results: Sixty-six eyes of 33 patients were included in the study. The sensitivity of UWF-FP for the detection of proliferative SCR was 100%, (95% confidence interval [CI95%] 76.8–100) for the retinal specialist and 100% (CI95% 71.5–100) for the ophthalmology resident. The specificity of UWF-FP for the detection of proliferative SCR was 100% (CI95% 92.7–100) for the retinal specialist and 98.1% (CI95% 89.7–100) for the ophthalmology resident. Conclusions: UWF-FP is a valuable exam for proliferative SCR screening, with excellent sensitivity and specificity and a good inter-grader agreement for ophthalmologists with various degree of skills, and is easy to use in a real-life setting.

## 1. Introduction

Sickle cell disease (SCD) is a complex multisystemic disease caused by a mutation in the β-globin gene resulting in the production of an abnormal hemoglobin variant that tends to polymerize and consequently cause erythrocytes deformation and rigidity. SCD is the most common monogenic disease worldwide and is predicted to become an increasing global health problem as a result of both the increase in life expectancy of the affected patients and the decrease of childhood mortality in developing countries [1,2]. In countries with a developed health care system, SCD has evolved into a chronic disorder. Until curative strategies become widely available, screening and management of clinical complications by a multidisciplinary team remain the centerpiece of medical care for this growing population of patients [3]. 

Ophthalmic complications of SCD can involve the orbit, the anterior segment, and the retina, the latter being the most frequent site. Because proliferative sickle cell retinopathy (SCR) can silently lead to vision threatening complications, an annual dilated retinal examination beginning at 10 years of age is recommended by experts [4]. In 1971, Goldberg studied the natural course of proliferative SCR and introduced an ophthalmoscopic and angiographic classification of SCR [5]. This classification is widely used in routine practice to assess SCR severity and to guide its management. 

The advent of ultrawide-field imaging (UWI) has allowed the acquisition of 200° images of the retina, with different modalities available, such as pseudocolor confocal scanning laser ophthalmoscopy (cSLO) ultrawide-field fundus photography (UWF-FP), ultrawide-field green-wavelength fundus autofluorescence (UWF-FAF), ultrawide-field fluorescein angiography (UWF-FA), and ultrawide-field indocyanine-green angiography (UWF-ICGA). Over the last decade, it has become a standard of care for various retinal pathologies, in particular for vascular diseases such as diabetic retinopathy, vein occlusion and SCR [6]. Both UWF-FP and UWF-FA provide a good visualization of peripheral vascular abnormalities occurring in SCR [7,8]. However, there is no clear recommendation regarding the need of fluorescein angiography for the follow-up and management of SCR. 

The aim of this study is to determine the sensitivity and specificity of UWF-FP for the detection and staging of SCR compared to UWF-FA. 

## 2. Experimental Section

### 2.1. Population

This study had the approval of the Ethics Committee of the Federation France Macula and adhered to the tenets of the Declaration of Helsinki. Written informed consent was obtained from all participants. This retrospective, monocentric study included patients with genetically confirmed SCD undergoing systematic ophthalmological evaluation or presenting with visual symptoms in the Créteil University Eye Clinic, France, between 22 May 2017 and 11 February 2019. Included patients had undergone UWF-FP and UWF-FA on the same day. Patients with a history of previous retinal photocoagulation or other confounding retinal vascular disease were excluded from the study.

### 2.2. Image Acquisition and Image Analysis

UWF-FP and UWF-FA were performed using Optos 200Tx (Optos PLC, Dunfermline, Scotland, UK). For each eye, several images were taken, and the analysis was based on the picture allowing the best visualization of the retinal periphery. Anonymized images were presented randomly on the Optos Viewer screen. UWF-FP and UWF-FA pictures were independently analyzed by a retinal specialist and an ophthalmology resident. Independent graders were allowed to adjust magnification, brightness, filters, and contrast of the images on the Optos viewer screen. Eyes were staged according to Goldberg classification of SCR: stage I corresponding to peripheral arteriolar occlusions, stage II corresponding to peripheral arteriolar-venular anastomoses, stage III corresponding to neovascular and fibrous proliferations, stage IV corresponding to vitreous hemorrhage, and stage V corresponding to retinal detachment [9]. Eyes with no retinopathy were graded as Goldberg Stage 0. For further analysis, Goldberg Stage 2 and below eyes (including salmon-patch hemorrhages, iridescent spots, black sunbursts, vascular tortuosity) were considered as non-proliferative and Goldberg Stage 3 and above eyes (including “sea fan” neovascularization, vitreous hemorrhage, and retinal detachment) were considered as proliferative. 

In case of disagreement between the two independent graders about the proliferative status, UWF-FA and UWF-RP were assessed by a third independent masked retinal specialist grader (A.M) for Goldberg stage. 

### 2.3. Statistical Analysis

Retinal specialist staging on UWF-FA was used for statistical tests on the cohort characteristics. Qualitative variables were expressed in percentages, and quantitative variables were expressed by their mean with standard deviation. Sensitivity and specificity of UWF-FP were calculated on the basis of the proliferative or non-proliferative status using UWF-FA as the gold standard. Cohen’s kappa calculation was used to evaluate the agreement between the independent graders and the imaging techniques. Fisher test was used to compare the distribution of a categorical variable in different groups. Kruskal–Wallis test was used to compare the distribution of a quantitative variable in different groups; *p*-value < 0.05 was retained as significant. STATA 13.0/SE (StatCorp, USA) was used for statistical tests. 

## 3. Results

### 3.1. Demographic Results

Sixty-six eyes of 33 patients (15 males and 18 females), aged 14–68 years (mean 27.70 ± 11.69 years old) were included in the study. Three out of 66 eyes were excluded because of pre-existing retinal photocoagulation. UWF-FP and UWF-FA images of 21 eyes from sickle cell hemoglobin C (HbSC) patients (33.33%), 38 eyes from sickle cell Hb S (HbSS) patients (60.32%), and 4 eyes from HbS β-thalassemia (HbS β-thal) patients (6.35%) were analyzed by the two graders.

On the basis of the retinal specialist staging on UWF-FA, the prevalence of proliferative SCR was 22.2% (95% confidence interval [CI95%] 12.7–34.5), and each stage was distributed within the study cohort as follows: 4 patients with stage 0 (6.3%), 27 patients with stage 1 (42.9%), 18 patients with stage 2 (28.6%), 13 patients with stage 3 (20.7%), 1 patient with stage 4 (1.6%), and 0 patient with stage 5 (0%). Table 1 summarizes the cohort characteristics.

### 3.2. Sensitivity and Specificity of UW-FP

**Figure 1:**
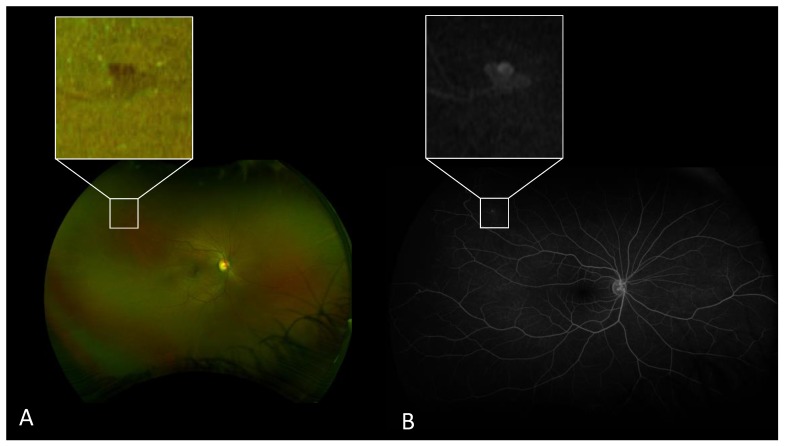
(**A**) Ultrawide-field fundus photography (UWF-FP) and (**B**) ultrawide-field fluorescein angiography (UWF-FA) of one of the three cases of inter-grader disagreement regarding the proliferative status. (**A**) UWF-FP shows peripheral arteriolar occlusions associated with a single red-colored lesion located in the superior temporal quadrant. Zooming on the lesion reveals the presence of small vascular loops suggestive of a nascent “sea fan” neovascularization. (**B**) UWF-FA and the corresponding zoom on the doubtful lesion confirms the presence of a nascent “sea fan” neovascularization.

The sensitivity of UWF-FP for the detection of proliferative SCR was 100% (CI95% 76.8–100) for the retinal specialist and 100% (CI95% 71.5–100) for the ophthalmology resident. The specificity of UWF-FP for the detection of proliferative SCR was 100% (CI95% 92.7–100) for the retinal specialist and 98.1% (CI95% 89.7–100) for the ophthalmology resident. For one eye, the ophthalmology resident detected a proliferative stage on the UWF-RP and a non-proliferative stage on the UWF-FA (Figure 1). 

### 3.3. Inter-Method Agreement

Agreement between UWF-FA and UWF-FP for the detection of proliferative SCR was 100% (kappa coefficient: 1.00) for the retinal specialist and 98.41% (kappa coefficient: 0.95) for the ophthalmology resident (Table 2). Agreement between UWF-FA and UWF-FP for SCR staging was 82.54 (kappa coefficient: 0.76) for the retinal specialist and 63.49% (kappa coefficient: 0.51) for the ophthalmology resident (Table 2). Table 3 summarizes the inter-method differences in Goldberg staging (UWF-FP compared to UWF-FA) for both graders. In 10 cases, the retinal specialist understated the stage by 1 stage when using UWF-FP with respect to UWF-FA. The ophthalmology resident understated the stage in 14 cases by 1 stage and in 2 cases by 2 stages when using UW-FP with respect to UWF-FA. 

### 3.4. Inter-Grader Agreement

Inter-grader agreement for the detection of proliferative SCR was of 95.24% on UWF-FA (kappa coefficient: 0.85) and 96.83% on UWF-FP (kappa coefficient: 0.90) (Table 2). There were three cases of disagreement concerning the proliferative status between the two graders, which were all graded non-proliferative by the ophthalmology resident and proliferative by the retinal specialist (Figure 1). These three cases were subsequently graded by the independent masked retinal specialist grader (A.M) as stage 3 on both UWF-FP and UWF-FA.

For the staging of SCR, inter-grader agreement was 52.38% (kappa coefficient: 0.36) on UWF-FP and 53.97% (kappa coefficient: 0.37) on UWF-FA (Table 2). Table 4 summarizes the differences in Goldberg staging on UWF-FA for the ophthalmology resident compared to the retinal specialist, considered as the reference. 

### 3.5. SCR Stages and SCD Genotype

Mean SCR stage (*p* = 0.004) and SCR stages distribution (*p* = 0.029) varied significantly between the different genotype groups (Table 1 and Table 5). Stage 1 was more frequent in the HbSS group (84% versus 16%), whereas stages 3 (52.85% versus 46.15%) and 4 (100% versus 0%) were more frequent in the HbSC group. 

There was no statistically significant difference of proliferative SCR between the HbSC group and the HbSS group (57.14% versus 42.86%, *p* = 0.065).

## 4. Discussion

This study emerged from a routine clinical problem which is the acceptability of using UWF-FP for the screening of SCD patients. To the best of our knowledge, this is the first study that calculates the sensitivity and specificity of UWF-FP for SCR screening by ophthalmologists with varying degrees of expertise. In this study, we evaluated the diagnostic performance of UWF-FP on 63 eyes of 33 patients with SCD using UWF-FA as the reference exam. We demonstrated that UWF-FP has excellent sensitivity and specificity for the detection of proliferative SCR for both retinal specialist (sensitivity 100%, specificity 100%) and ophthalmology resident (sensitivity 100%, specificity 98.1%). Agreement between UWF-FP and UWF-FA concerning Goldberg staging was high for the retinal specialist (kappa coefficient: 0.76) and mild for the ophthalmology resident (kappa coefficient: 0.51), hence reflecting a learning curve for the evaluation of peripheral vascularization on UWF-FP. However, for both independent graders, UWF-FP tends to underestimate the Goldberg stage compared to UWF-FA, predominantly for stages 1 and 2. This result is consistent with a previous study by Han et al. in which UWF-FA resulted in the identification of higher stages than UWF-FP and clinical examination [8]. Moreover, as shown in Figure 1, the use of zoom and a green filter on the viewer is primordial to enhance the detection of vascular modifications. However, despite careful examination, ischemic areas and peripheral anastomoses can be vague on UWF-FP. Inter-grader agreement was excellent for the detection of proliferative SCR on both imaging modalities. A careful analysis of the three cases of disagreement between the two graders revealed a very early stage of neovascularization, for which the indication of retinal photocoagulation would have been controversial, knowing the possibility of spontaneous regression of such lesions [10]. Otherwise, inter-grader agreement for Goldberg classification was fair on both imaging modalities (kappa coefficient 0.36 for UWF-FP and 0.37 for UWF-FA). The different level of expertise of the independent graders can explain this result. Most of the disagreement involved an undervaluation of stages 1 and 2 by the ophthalmology resident, illustrating the difficulty for an inexperienced ophthalmologist to distinguish between these two stages. However, our results are consistent with those of Han et al. regarding the mild inter-grader agreement for Goldberg classification on UWF-FP involving stages 1 and 2 [8]. 

The study limitations include the relatively small sample size, the high proportion of patients with severe SCD managed in our reference center for SCD, as well as the absence of stage 5 lesions from our study cohort. Furthermore, this was a retrospective study with varying image quality, reflecting real-life practice. The small number of graders may limit the extent to which the survey findings can be generalized to all the ophthalmologists. However, we intentionally chose two graders with a high difference in expertise level. This way, our results may be more representative of a wide range of ophthalmologists with various degrees of skills.

Conventional fluorescein angiography (FA) has been considered as the gold standard for the detection and classification of SCR for several decades [9]. UWF-FA has been compared to conventional FA for the analysis of various retinal pathologies, such as diabetes or retinal vein occlusion [11,12,13,14,15], revealing a better visualization of the peripheral retina, while reducing the need of patient cooperation, as well as patient discomfort. Moreover, UWI offers the further advantage of being compatible with all type of patients, pediatric or uncooperative, with few light flashes and no pupil dilatation [16].

The use of UWI in SCR patients was first analyzed in 2011 by Cho et al., who demonstrated that UWF Imaging offers a better visualization of peripheral vascular changes compared to clinical examination, with a significant impact on management decisions [7]. Conversly, Han et al. considered that the higher lesion stages detected on UWI compared to clinical examination did not modify the therapeutic strategies [8]. On the basis of these studies, we decided to use UWF-FA as the reference exam to evaluate UWF-FP. 

The detection of proliferative SCR has a direct impact in terms of therapeutic management, as it corresponds to the classical indication for retinal photocoagulation [17]. The impact of the disagreements between UWF-FP and UWF-FA regarding Goldberg stages is mild, involves mainly non-proliferative stages, and does not modify the therapeutic management. Nonetheless, UWF-FP appears to be less accurate than UWF-FA to evaluate the risk of progression and to adjust the interval of follow-up. This finding may impact the prognosis of non-compliant patients lost to follow-up. However, the indication of FA in early stages of SCR should be weighed against the potential risk of anaphylactic shock and other side effects of fluorescein injection [18,19]. Recently, other indicators of risk of progression have been described using non-invasive imaging techniques, such as optical coherence tomography (OCT) and OCT angiography [20,21]. Such indicators could be used in complement to UWF-FP to evaluate the prognosis and severity of SCR and to adjust the interval of follow-up. However, UWF-FA remains the reference exam allowing an excellent visualization of ischemic areas, peripheral anastomoses, and leakage associated with neovascularization. It can be particularly useful in ambiguous cases of early neovascularization and before retinal photocoagulation to target ischemic areas.

In conclusion, our study confirms that UWF-FP tends to underestimate Goldberg stages compared to UWF-FA, in particular stages 1 and 2. UWF-FP is a valuable exam for proliferative SCR screening, with excellent sensitivity and specificity and a good inter-grader agreement for ophthalmologists of varying degrees of skills, and is easy to implement in a real-life setting.

## Figures and Tables

**Table 1:** Demographic and clinical characteristics of the cohort. HbSS: sickle cell hemoglobin S, HbSC: sickle cell Hb C, HbS β-thal: HbS β-thalassemia.

	All Patients *n* = 63 Eyes, 33 Patients		HbSS Group *n* = 38 Eyes, 20 Patients		HbSC Group *n* = 21 Eyes, 11 Patients		HbS β-thal Group *n* = 4 Eyes, 2 Patients		*p*-Value
	Mean or *n*	SD or Percent	Mean or *n*	SD or Percent	Mean or *n*	SD or Percent	Mean or *n*	SD or Percent	
Age	27.70	11.69	25.75	8.72	28.82	10.12	41	38.18	0.70
Male sex	15	45.45	8	40	6	54.54	1	50	0.73
Goldberg stage	1.68	0.93	1.55	0.80	2.14	0.96	0.5	0.58	0.004
stage 0	4	6.35	1	2.63	1	4.76	2	50.00	N/A
stage 1	27	42.86	21	55.26	4	19.05	2	50.00	N/A
stage 2	18	28.57	10	26.32	8	38.10	0	0	N/A
stage 3	13	20.63	6	15.79	7	33.33	0	0	N/A
stage 4	1	1.59	0	0	1	4.76	0	0	N/A
stage 5	0	0	0	0	0	0	0	0	N/A

**Table 2:** Inter-grader and inter-method agreement for the detection of proliferative sickle cell retinopathy (SCR) and SCR staging according to Goldberg classification.

**Inter-Method**	**Retinal Specialist**		**Ophthalmology Resident**	
	**Agreement (%)**	**Kappa Coefficient**	**Agreement (%)**	**Kappa Coefficient**
Proliferative SCR	100	1	98.41	0.9468
SCR staging	82.5	0.7561	63.49	0.5116
**Inter-Grader**	**UWF-FP**		**UWF-AF**	
	**Agreement (%)**	**Kappa Coefficient**	**Agreement (%)**	**Kappa Coefficient**
Proliferative SCR	96.83	0.9032	95.24	0.8508
SCR staging	52.38	0.3647	53.97	0.3669

**Table 3:** Differences in Goldberg stages using UWF-FP and UWF-FA.

Expertise Level	Stages Difference UWF-FP vs. UWF-FA	Eyes (%)	Stages (eyes)
**Retinal specialist**	+ 1 stage difference	1 (1.6)	2-1 (1)
	No stage difference	52 (82.5)	-
	− 1 stage difference	10 (15.9)	0-1 (6)
			1-2 (4)
**Ophthalmology resident**	+ 2 stages difference	2 (3.2)	2-0 (2)
	+ 1 stage difference	5 (7.9)	1-0 (1)
			2-1 (3)
			3-2 (1)
	No stage difference	40 (63.5)	
	− 1 stage difference	14 (22.2)	0-1 (7)
			1-2 (7)
	− 2 stages difference	2 (3.2)	0-2 (2)

**Table 4:** Ophthalmology resident Goldberg staging compared to retinal specialist Goldberg staging on UWF-FA.

Stages Difference	Eyes (%)	Stages (Eyes)
+ 1 stage difference	7 (11.11)	2-1 (7)
No stage difference	34 (53.97)	-
− 1 stage difference	21 (33.33)	0-1 (9)
		1-2 (10)
		2-3 (2)
− 2 stages difference	1 (1.59)	3-1 (1)

**Table 5:** SCR staging according to sickle cell disease (SCD) genotype.

Goldberg Stage	Genotype			Total
	SS (%)	SC (%)	Sβthal (%)	
0	1 (25)	1 (25)	2 (50)	4
1	21 (77,78)	4 (14,81)	2 (7,41)	27
2	10 (55,56)	8 (44,44)	0 (0)	18
3	6 (46,15)	7 (53,85)	0 (0)	13
4	0 (0)	1 (100)	0 (0)	1
Total	38 (60,32)	21 (33,33)	4 (6,35)	63 (100)
